# Comparison of Psychological Symptoms and Serum Levels of Neurotransmitters in Shanghai Adolescents with and without Internet Addiction Disorder: A Case-Control Study

**DOI:** 10.1371/journal.pone.0063089

**Published:** 2013-05-03

**Authors:** Hong-Xia Zhang, Wen-Qing Jiang, Zhi-Guang Lin, Ya-Song Du, Alasdair Vance

**Affiliations:** 1 Department of Psychological Medicine, Zhongshan Hospital, Fudan University, Shanghai, China; 2 Department of Child and Adolescent Psychiatry, Shanghai Mental Health Center, Shanghai Jiao Tong University School of Medicine, Shanghai, China; 3 Department of Biological Psychiatry, Shanghai Mental Health Center, Shanghai Jiao Tong University School of Medicine, Shanghai, China; 4 Academic Child Psychiatry Unit, Royal Children’s Hospital, University of Melbourne, Melbourne, Victoria, Australia; Federal University of Rio de Janeiro, Brazil

## Abstract

**Background:**

Internet addiction disorder (IAD) is now recognized internationally and is known to be linked with academic and social impairment. To date, we know little about its associated main biological factors. This study aimed to collect a carefully defined group of adolescents with IAD and an age- and gender-matched typically developing comparison group. We hypothesized that the young people with IAD would have higher rates of self-reported anxiety and depressive symptoms, have altered levels of peripheral blood dopamine, norepinephrine and serotonin. In addition, we hypothesized the hours spent online are correlated with the severity of depression and anxiety among these young people with IAD.

**Methodology/Principal Finding:**

A cross-sectional study of 20 adolescents who met Beard’s criteria for IAD and 15 typically developing adolescents (comparison group) was conducted. All the participants completed the Self Rating Depression Scale (SDS), Self Rating Anxiety Scale (SAS), and the Screen for Child Anxiety Related Emotional Disorders (SCARED). Peripheral blood dopamine, serotonin and norepinephrine were assayed. The mean level of norepinephrine was lower in the IAD group than that in the typically developing participants, while dopamine and serotonin levels did not differ. The SDS, SAS and SCARED symptom scores were increased in the adolescents with IAD. A logistic regression analysis revealed that a higher SAS score and lower level of norepinephrine independently predicted IAD group membership. There was no significant correlation between hours spent online and scores of SAS/SDS in IAD group.

**Conclusions/Significance:**

Increased self-reported anxiety and lower peripheral blood norepinephrine are independently associated with IAD.

## Introduction

Internet addiction disorder (IAD) has arisen with the increased popularity of the internet: indeed, point prevalence rates are known to have increased in developing and developed countries [1]–[3]. Functional impairments in academic, social, family and occupational domains have been documented and linked to IAD [2], [4]. A number of factors have been proffered such as younger age of internet use, increased anxiety and increased depressive symptoms and/or disorders [5]–[7]: higher scores on the Beck Depression Inventory (BDI) [6] or the Center for Epidemiological Studies Depression Scale (CES-D) [7] are associated with IAD. Further, higher emotional disorder scores on the Strengths and Difficulties Questionnaire, higher levels of anxiety and increased suicidal ideation have been reported in young people with IAD [8]–[10].

Currently, the main biological factors related to IAD remain unclear [6]. Likely factors include the imbalance of the functional levels of dopamine (DA), serotonin (5–HT), and/or norepinephrine (NE), which are associated with the onset of mood and anxiety disorders as is the imbalance of serotonin and norepinephrine neuronal axon regeneration [11]–[15]. Further, a reduced functional serotonin turnover rate has been linked to major depressive disorder and may be implicated in IAD [16]. We hypothesized that the young people with IAD would have higher rates of self-reported anxiety and depressive symptoms and altered levels of peripheral blood dopamine, norepinephrine and serotonin.

Tonioni et al. [17] have identified a relationship between hours spent online and depression/anxiety levels, we hypothesized the hours spent online may also be correlated with scores of SAS/SDS among the young people with IAD.

## Materials and Methods

### Participants

20 adolescent students with IAD, according to Beard’s criteria [17], were recruited from the outpatient department of the Shanghai Mental Health Center at Shanghai Jiao Tong University School of Medicine from July 2008 to January 2010. These students were spending approximately 33.8 (16.8) hours per week using the internet online. They were all preoccupied with the Internet (thinking about previous online activity or anticipating their next online session); needing to use the Internet for increasing periods of time to be sated; unable to control, cut back, or stop their Internet use; restless, moody, depressed, and/or irritable when their Internet use was cut down or stopped; and staying online longer than originally intended. In addition, they had manifest at least one of the following three symptoms: risked the loss of a significant relationship, job, educational or career opportunity because of their Internet use; lied to family members or others to conceal the extent of their Internet use; and/or used the Internet as a way of escaping from problems or of relieving a dysphoric mood. They were considered functionally impaired if they had underachieved academically, manifest school refusal behavior and/or been disciplined by authority figures (teachers and/or parents) because of their Internet overuse. Students were excluded if they had evidence of any comorbid medical disorder, pre-existing psychiatric disorder and/or were taking any psychoactive medication.

Typically developing adolescent volunteers, matched for age and gender, from the same socio-demographic neighborhood (middle school in Shanghai) without medical or psychiatry disorder, alcohol and/or substance use were recruited as healthy control participants. Informed consent was obtained from all participants and their legal guardians. There were 18 boys and 2 girls (mean age of 16.8±1.8 years) in the IAD group and 13 boys and 2 girls (mean age of 18.1±2.7 years) in the typically developing group.

### Ethics Statement

This study was a part of a large research that focused on adolescent behavior disorders. The latter was approved by the Institute Review Board of Shanghai Mental Health Center. The study was conducted in Shanghai only, not outside China. Participants and their legal guardians attended the Shanghai Mental Health Center at Shanghai Jiao Tong University School of Medicine for a two hour session with breaks as needed. Written informed consent was obtained at the beginning of the session, after tests were clearly explained to participants and their legal guardians.

### Measures

Beard’s Diagnostic Questionnaire for Internet Addiction [18]: 8 items in all, with a dichotomous (Yes/No) Likert scale. IAD is diagnosed when all of the first 5 items are met with at least one of the following 3 items met.

Self Rating Depression Scale (SDS) [19]: 20 items with a four-point Likert scale. A higher score indicates more severe depressive symptoms. Validity and reliability are adequate in the People’s Republic of China.

Self Rating Anxiety Scale (SAS) [20]: 20 items with a four-point Likert scale. A higher score indicates more severe anxiety symptoms. Validity and reliability are adequate in the People’s Republic of China.

Screen for Child Anxiety Related Emotional Disorders (SCARED) [21], [22]: 41 items in all with a three-point Likert sale supporting five factors: ‘somatic/panic’, ‘generalized anxiety’, ‘separation anxiety’, ‘social anxiety’ and ‘school anxiety’. The higher the score, the higher the level of a given anxiety factor in a child. Validity and reliability are adequate in the People’s Republic of China.

### Biochemistry Tests

For each participant, 5 ml of venous blood was extracted using a heparin anticoagulation vacuum tube, kept in a cold state with light avoided. The levels of DA and NE in serum were measured using ELISA (enzyme linked immuno-sorbent assay), and the level of 5-HT in peripheral blood platelets was measured with HPLC (high performance liquid chromatography).

### Procedure

Participants and their legal guardians attended the Shanghai Mental Health Center at Shanghai Jiao Tong University School of Medicine. Written informed consent was obtained. All tests were administered by registered medical practitioners and the data derived from them entered onto the computer database.

### Statistical Analysis

Data were analyzed using the Statistical Package for Social Sciences (SPSS), version 16.0 to compare the IAD and typically developing groups. Variables that were normally distributed, defined by the Kolmogorov-Smirnov test, or that could be converted to a normal distribution were compared using independent-sample *t* tests. Non-parametric data were compared using the Mann-Whitney *U* test. Pearson product-moment correlation coefficients were calculated for variables that differed between the IAD and typically developing groups. Also, these variables were entered into a binary logistic regression analysis to determine which variables independently predicted IAD group membership. Correlation between hours spent online and scores of SAS/SDS in IAD group was determined by Pearson *r*.

## Results

### Level of Monoamine Neurotransmitters in Plasma

The mean level of NE in the IAD group was lower than that in the typically developing group [(345±68) pg/ml and (406±76) pg/ml, respectively, *t* = 2.515, *p* = 0.017]. There was no significant difference in the levels of DA or 5-HT between the two groups (Table 1).

**Table 1:** The level of 5-HT, NE and DA in IAD and control groups.

	IAD (n = 20)	Control (n = 15)	*t*-value	*p*-value
5-HT (ng/10^9^ platelet)	202±34	217±48	1.109	0.275
NE (pg/ml)	345±68	406±76	2.515	0.017
DA (pg/ml)*	20.9 (6.6, 75.4)	21.9 (17.5, 41.6)	–	–
Logarithm of DA	1.53±0.38	1.52±0.40	0.100	0.921

Abbreviation. IAD: Internet addiction disorder; 5-HT: serotonin; NE: norepinephrine; DA: dopamine.

* median (interquartile distance), other data in mean ± SD.

### Self Reported Emotional Symptoms

The SDS, SAS and SCARED scores in the IAD group were all significantly higher than those in the typically developing group (Table 2).

**Table 2:** Comparison of scores of self reported emotional symptoms between IAD and control groups.

	IAD (n = 20)	Control (n = 15)	*t*-value	*p*-value
	(Mean±SD)	(Mean±SD)		
Score of SDS	53.29±9.20	40.87±5.49	4.700*	<0.001
Score of SAS	66.18±11.42	50.67±6.74	4.741*	<0.001
Score of SCARED	22.76±11.96	9.27±8.31	3.659	0.001

Abbreviation. IAD: Internet addiction disorder; SDS: Self Rating Depression Scale; SAS: Self Rating Anxiety Scale; SCARED: Screen for Child Anxiety Related Emotional Disorders.

* heterogeneity of variance, modified *t*-value.

### Correlation of Self Reported Emotional Symptoms with NE Level

The Pearson product-moment correlation coefficients for the IAD and typically developing groups ranged between −0.26 to −0.29 for the level of NE and the scores of SDS, SAS and SCARED (*r* = −0.263, −0.269 and −0.294, respectively).

### Logistic Regression Analysis

The independent variables entered into the logistic regression were NE level and the SDS, SAS and SCARED scores. Age and gender were also considered as independent variables. Two variables remained in the regression equation: the SAS score (V1) and the NE level (V2) (Table 3). The overall correct percentage was 80.0% (regression equation: logit(*P*) = −14.729+0.475×V1−0.031×V2).

**Table 3:** Results of logistic regression of the NE level and the severity of self reported emotional symptoms with the diagnosis of IAD or not (20 adolescences with IAD and 15 controls).

	B	Wald	*p*	OR	95%CI
Score of SAS	0.475	4.269	0.039	1.607	1.025–2.521
NE level	−0.031	3.530	0.060	0.970	0.939–1.001

Abbreviation. IAD: Internet addiction disorder; SAS: Self Rating Anxiety Scale; NE: norepinephrine.

### Correlation of Hours Spent Online and Scores of SAS/SDS Among Adolescents with IAD

Among the 20 young people with IAD, the correlation coefficient of hours spent online per week to the score of SAS was not statistically significant (*r = *0.015, *p = *0.955), nor was that to the score of SDS (*r = *0.015, *p = *0.954).

## Discussion

The finding that adolescents with IAD have elevated anxiety and depressive symptoms is consistent with previous work: Bernardi et al. [23] found that 30% of young people with IAD had clinically significant levels of anxiety. Other studies have noted the greater-than-chance association of IAD with depressive disorders [7], [9], [24], [25] and the increased likelihood that young people with a depressive disorder may develop IAD [26], [27]: importantly, our previous randomized controlled trial found that adolescents with IAD had improved anxiety and depressive symptoms after cognitive behavioral therapy [28].

Interestingly, although altered DA, NE and 5-HT functional activity has been linked with clinically significant anxiety and depressive symptoms, we found that only the level of NE was lower and self reported anxiety higher in the IAD group compared to the typically developing group. Further, a lower level of NE correlated slightly with increased anxiety and depressive symptoms. So, indeed mood and predominantly anxiety problems in adolescents with IAD may be associated with altered monoamine functional activity: however, 5-HT and DA were not implicated, suggesting there may be a NE-specific biological factor associated with IAD in adolescence. One important implication of this may be that dopamine mediated reinforcement of addictive behavior is not associated with IAD, as in other forms of addiction [29]. However, given that NE is a metabolic product of DA, further systematic examination is needed. Zhu et al. [30] have recently noted that changes in peripheral blood NE level may be associated with effective treatment of IAD and associated depressive and anxiety symptoms. Again, future controlled trials are needed.

Although previous studies [17], [31] suggest a relation between hours spent online and depression/anxiety levels, we did not found such positive relation in this study. To explain the difference, there may be some other factors deserved further study besides the different assessment instructions for emotion (SCL-90 in the previous two studies and SAS, SDS in our study).

There are several limitations in this study that constrain the interpretation of the results. First, the small sample size may lead to increased type 1 and 2 error rates. Second, the limited age range and gender distribution of the sample mean that inferences about developmental stage and gender can’t be drawn. Third, the lack of longitudinal data means no causal inferences can be made from the significant associations presented. Clearly, larger longitudinal samples of boys and girls, across childhood, adolescence and young adulthood, carefully defined for IAD and key comorbid disorders, obtained from multiple centers would address these limitations. In addition, future examination of improved NE levels in controlled treatment trials is needed.
